# Effect of Composition on the Growth of Single Crystals of (1−x)(Na_1/2_Bi_1/2_)TiO_3_-xSrTiO_3_ by Solid State Crystal Growth

**DOI:** 10.3390/ma12152357

**Published:** 2019-07-24

**Authors:** Phan Gia Le, John G. Fisher, Won-Jin Moon

**Affiliations:** 1 School of Materials Science and Engineering, Chonnam National University, 77 Yongbong-ro, Buk-gu, Gwangju 61186, Korea; 2 Korea Basic Science Institute, Gwangju Center, 77 Yongbong-ro, Buk-gu, Gwangju 61186, Korea

**Keywords:** lead-free piezoelectric, (Na_1/2_Bi_1/2_)TiO_3_, single crystal growth, grain growth, micro-structure

## Abstract

The (1−x)(Na_1/2_Bi_1/2_)TiO_3_-xSrTiO_3_ (NBT-100xST) system is a possible lead-free candidate for actuator applications because of its excellent strain vs. electric field behaviour. Use of single crystals instead of polycrystalline ceramics may lead to further improvement in piezoelectric properties but work on single crystal growth in this system is limited. In particular, the effect of composition on single crystal growth has yet to be studied. In this work, single crystals of (NBT-100xST) with x = 0.00, 0.05, 0.10 and 0.20 were grown using the method of Solid State Crystal Growth. [001]-oriented SrTiO_3_ single crystal seeds were embedded in (NBT-100xST) ceramic powder, which was then pressed to form pellets and sintered at 1200 °C for 5 min–50 h. Single crystal growth rate, matrix grain growth rate and sample microstructure were examined using scanning and transmission electron microscopy. The results indicate that the highest single crystal growth rate was obtained at x = 0.20. The mixed control theory of grain growth is used to explain the single crystal and matrix grain growth behaviour.

## 1. Introduction

(Na_1/2_Bi_1/2_)TiO_3_-based materials are candidate lead-free piezoelectric materials [1,2]. (Na_1/2_Bi_1/2_)TiO_3_ (NBT) is a perovskite material with rhombohedral symmetry at temperatures lower than 255 °C, tetragonal symmetry at temperatures between 255–540 °C, and cubic symmetry at temperatures above 540 °C [3,4,5,6,7]. (Na_1/2_Bi_1/2_)TiO_3_ also has a depolarization temperature T_d_ = 186 °C and a temperature of the maximum value of relative permittivity T_m_ = 320 °C [8]. The position of T_d_, the rhombohedral-tetragonal phase transition temperature T_R-T_ and T_m_ can be altered by adding dopants or by forming solid solutions with other perovskite compounds [8,9,10,11]. (Na_1/2_Bi_1/2_)TiO_3_ also exhibits strong ferroelectricity with remnant polarization of P_r_ = 38 μC/cm^2^, but the high coercive field of 7 kV/mm makes the poling process difficult [12].

To improve the electrical properties, (Na_1/2_Bi_1/2_)TiO_3_ is often combined with a compound with tetragonal or cubic symmetry e.g., BaTiO_3_, SrTiO_3_ or (Bi_1/2_K_1/2_)TiO_3_. These solid solutions exhibit a morphotropic phase boundary (MPB) that leads to improved piezoelectric properties. The MPB in these solid solutions is found at 6–11 mol % BaTiO_3_ in the (Na_1/2_Bi_1/2_)TiO_3_-BaTiO_3_ system, 16–22 mol % (K_1/2_Bi_1/2_)TiO_3_ in the (Na_1/2_Bi_1/2_)TiO_3_-(K_1/2_Bi_1/2_)TiO_3_ system and 20–30 mol % SrTiO_3_ in the (Na_1/2_Bi_1/2_)TiO_3_-SrTiO_3_ system [2,11,13,14,15,16]. At the MPB compositions, the ferroelectric and piezoelectric properties are much improved but this is accompanied by a decrease in T_d_, which leads to a more narrow operating temperature range.

In the (1−x)(Na_1/2_Bi_1/2_)TiO_3_-xSrTiO_3_ system, T_m_, T_d_, and T_R-T_ shift towards lower temperatures with increasing amounts of SrTiO_3_ [9,11,12]. In particular, at x ≥ 0.2 T_d_ and T_R-T_ converge. Compositions in this region show that extraordinary strains can be induced by an electric field [2,9,17]. Hiruma et al. showed the existence of a rhombohedral/pseudocubic MPB at x = 0.26 to 0.28, a giant strain = 0.29% and an inverse piezoelectric coefficient $d_{33}^{*}$ = 488 pm/V at x = 0.28 [9]. This property makes (1−x)(Na_1/2_Bi_1/2_)TiO_3_-xSrTiO_3_ compositions attractive for stack actuator applications. Krauss et al. found that the strain reaches a maximum of 0.29% at x = 0.25 [12]. Acosta et al. reported that an electric field could induce a phase transition between relaxor and ferroelectric phases for the composition with x = 0.25, with an inverse piezoelectric coefficient $d_{33}^{*}$ = 600–700 pm/V at an electric field of 4 kV/mm [17].

In the previous works, polycrystalline ceramic (1−x)(Na_1/2_Bi_1/2_)TiO_3_-xSrTiO_3_ solid solutions were studied. Numerous studies have shown the improved piezoelectric properties of NBT-based single crystals [18,19,20,21]. Hence, single crystals of (1−x)(Na_1/2_Bi_1/2_)TiO_3_-xSrTiO_3_ are also expected to have improved properties. However, only limited work has been done on growing single crystals in this system [22,23]. Single crystals of (1−x)(Na_1/2_Bi_1/2_)TiO_3_-xSrTiO_3_ compositions are grown using the solid state crystal growth (SSCG) method in the present work [24,25]. In this method a single crystal (the seed crystal) is embedded in powder of the single crystal composition and sintered. A single crystal of the powder composition grows epitaxially on the seed crystal. This method uses conventional ceramic processing equipment and lower processing temperatures than flux-based methods [25,26]. Because the batch material does not have to be melted and recrystallized, evaporation of Bi_2_O_3_ and Na_2_O is reduced and chemical homogeneity is improved. Single crystals of (Na_1/2_Bi_1/2_)TiO_3_-BaTiO_3_, (Na_1/2_Bi_1/2_)TiO_3_-CaTiO_3_, 0.8(Na_1/2_Bi_1/2_)TiO_3_-0.2SrTiO_3_, 0.75(Na_1/2_Bi_1/2_)TiO_3_-0.25SrTiO_3_, (Na_1/2_Bi_1/2_)TiO_3_-Ba(Ti,Zr)TiO_3_ and (Na_1/2_Bi_1/2_)TiO_3_-BaTiO_3_-(K_0.5_Na_0.5_)NbO_3_ have been grown using this method [19,22,23,27,28,29].

In the SSCG method, the mean grain size of the ceramic matrix that surrounds the growing single crystal is important, as this affects the driving force for growth and therefore the growth rate of the single crystal [24,30,31]. Krauss et al. found that the matrix grain size became smaller in their (1−x)(Na_1/2_Bi_1/2_)TiO_3_-xSrTiO_3_ samples as the SrTiO_3_ amount increased [12]. Hence changes in composition may be expected to change the growth rate of single crystals of (1−x)(Na_1/2_Bi_1/2_) TiO_3_-xSrTiO_3_ grown by SSCG. In this work, the effect of composition on the matrix grain and single crystal growth behaviour of (1−x)(Na_1/2_Bi_1/2_)TiO_3_-xSrTiO_3_ will be studied in order to find the most suitable composition for single crystal growth.

## 2. Materials and Methods

(1−x)(Na_1/2_Bi_1/2_)TiO_3_-xSrTiO_3_ powders with x = 0.00, 0.05, 0.10 and 0.20 (NBT-100xST) are synthesized from Na_2_CO_3_ (99.5%, ACROS Organics, Geel, Belgium), Bi_2_O_3_ (99.9%, Alfa Aesar, Ward Hill, MA, USA)_,_ TiO_2_ (99.8%, Alfa Aesar, Ward Hill, MA, USA ) and SrCO_3_ (≥99.9%, Aldrich, St. Louis, MO, USA) starting materials by solid state reaction. For more details of the synthesis procedure please see ref. [23]. The calcined NBT-100xST powders are analyzed by X-ray diffraction (XRD, X’Pert PRO, PANalytical, Almelo, The Netherlands) with Cu K_α_ radiation, 2θ = 20°–80°, scan speed = 3° min^−1^ and 0.02° step size. The crystal structure and phase are identified with MDI Jade 6 (Materials Data Inc., Livermore, CA, USA). Calcined powders are planetary ball milled as before and examined by scanning electron microscopy (SEM, Hitachi S-4700, Tokyo, Japan). ImageJ v1.50a image analysis software (National Institute of Mental Health, Bethesda, MD, USA) is used to analyze particle size distributions from SEM micrographs.

[001]-oriented single crystals of SrTiO_3_ (MTI Corp., Richmond, CA, USA) of dimensions 5 mm × 5 mm × 0.5 mm are used as seed crystals. Seeds are buried in ~0.6 g of the NBT-100xST powders in a 10 mm steel die and hand-pressed into pellets, followed by cold isostatic pressing (CIP) at 1500 kg/cm^2^ (~147 MPa). Pellets are then placed in high purity alumina double crucibles with lids and sintered at 1200 °C for 5 min, 1, 3, 5, 10, 20 and 50 h (heating and cooling rates of 5 °C min^−1^). To reduce evaporation of Na and Bi, pellets are buried inside packing powder of the same composition during sintering. Sintered samples are vertically sectioned and polished to a 1 µm finish, followed by thermal etching. Pt-coated samples are observed by scanning electron microscopy (SEM, Hitachi S-4700, Tokyo, Japan) with attached energy dispersive X-ray spectrometer (EDS, EMAX energy EX-200, Horiba, Kyoto, Japan) with standardless quantification. ImageJ v1.50a image analysis software is used to analyze single crystal growth distance, mean matrix grain size and matrix grain size distribution from SEM micrographs.

For detailed observation of the matrix grain boundary and triple junction structure, NBT-0ST and NBT-10ST ceramic samples (without seed crystals) are sintered at 1200 °C for 10 h. Transmission electron microscopy (TEM) specimens are prepared by standard methods of sectioning, grinding, dimpling and ion milling (PIPS, Gatan Inc., Pleasanton, CA, USA). Specimens for transmission electron microscopy are examined in a TEM (FEI TECNAI F 20, FEI comp., Hillsboro, OR, USA) operated at an accelerating voltage of 200 kV.

## 3. Results

**Figure 1:**
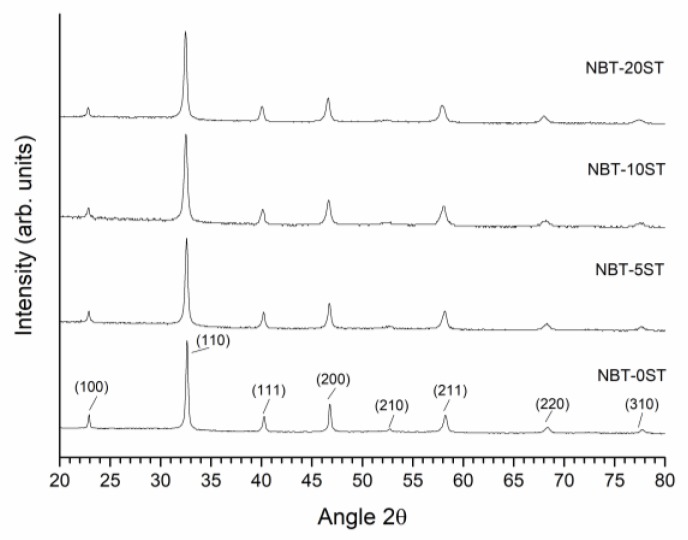
X-ray diffraction (XRD) patterns of (1−x)(Na_1/2_Bi_1/2_)TiO_3_-xSrTiO_3_ powders calcined at 850 °C for 3 h.

**Figure 2:**
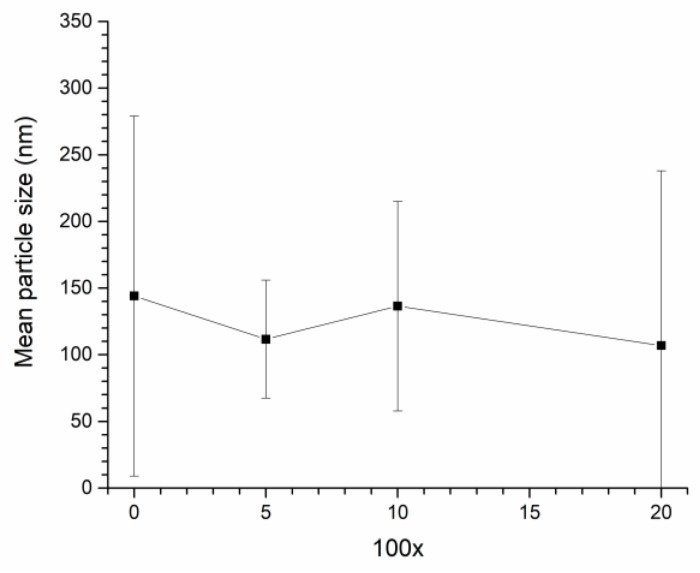
Mean particle size of (1−x)(Na_1/2_Bi_1/2_)TiO_3_-xSrTiO_3_ powders.

XRD patterns of calcined NBT-100xST powders are shown in Figure 1. All peaks in the XRD patterns were indexed with pdf card # 98-015-4342 in the ICDD database (rhombohedral symmetry, *R3c* space group). Secondary phases are not visible in the XRD patterns of the NBT-100xST powders. The mean particle size of NBT-100xST powders as a function of SrTiO_3_ content are shown in Figure 2. 

**Figure 3:**
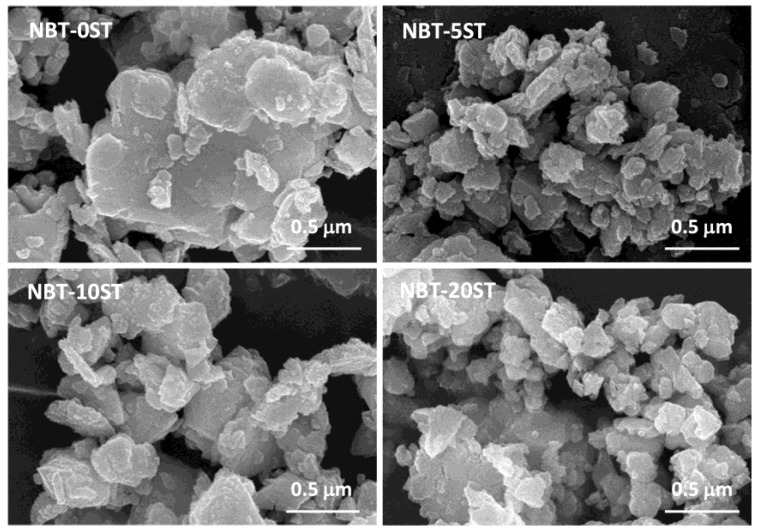
Scanning electron microscopy (SEM) micrographs of (1−x)(Na_1/2_Bi_1/2_)TiO_3_-xSrTiO_3_ powders.

To measure the size of a particle, the area of the particle is measured using the ParticleAnalyzer add-on for ImageJ and converted to an equivalent 2D spherical radius. For the case where a large particle is covered by several small particles (e.g., for the sample NBT-0ST in Figure 3), separate measurements are made for the area of the large and small particles (when the area of the large particle is measured, the small particles on top of it are ignored). If a particle is heavily obscured by other particles, it is not measured. Each data point is the mean value of at least 150 particles. Standard deviation is represented by the error bars. The mean particle size of the powders varies from ~105–145 nm. Mean particle size does not show a dependence on SrTiO_3_ content. The powders have broad particle size distributions as shown by the wide error bars. Figure 3 shows SEM micrographs of the NBT-100xST powders. The particles range in size from <100 nm to 300 nm, with some agglomerates of partially sintered particles.

**Figure 4:**
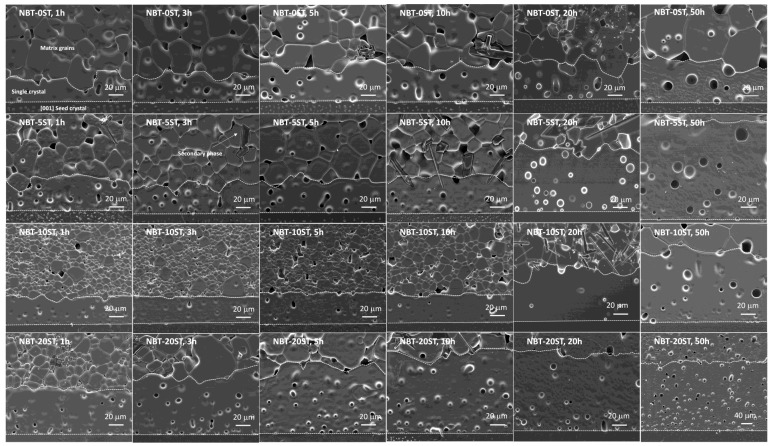
SEM micrographs of the (1−x)(Na_1/2_Bi_1/2_)TiO_3_-xSrTiO_3_ samples.

**Figure 5:**
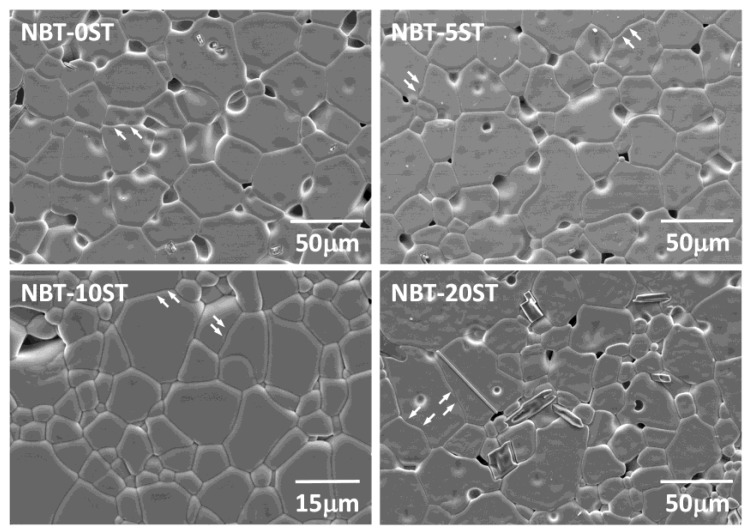
SEM micrographs of the matrix grains of (1−x)(Na_1/2_Bi_1/2_)TiO_3_-xSrTiO_3_ samples sintered at 1200 °C for 10 h.

Figure 4 shows SEM micrographs of the sintered NBT-100xST samples. Single crystal growth has taken place on the seed crystals. Matrix grain growth has also taken place. Both the single crystals and the surrounding matrix are porous. Seed crystal/single crystal and single crystal/matrix grain boundaries are marked by white dashed lines. Many of the samples appear to contain secondary phases in the matrix grains. According to EDS analysis, some of the secondary phase particles are Bi and Na-deficient compared to the matrix grains, while others are Bi-deficient and Ti-rich. The matrix grains in the NBT-10ST samples are noticeably smaller than those in the other samples. Figure 5 shows SEM micrographs of matrix grains in the samples sintered for 10 h. The grains are equiaxed. Micro-faceting is visible on some of the grain boundaries (marked with arrows).

EDS analysis of an NBT-20ST sample sintered for 10 h is shown in Table 1. Each value is the mean and standard deviation of five measurements. The single crystal was measured 5 times and 5 matrix grains were measured (one measurement each). The results have been normalized to a nominal Ti concentration of 20 at. %. The chemical composition of the single crystal and the matrix grains are similar. Both the single crystal and the matrix grains show some Na loss due to volatilization during sintering, as previously found [23]. The oxygen content is much lower than the nominal value, due to the difficulty in measuring the concentration of low atomic number elements accurately.

**Figure 6:**
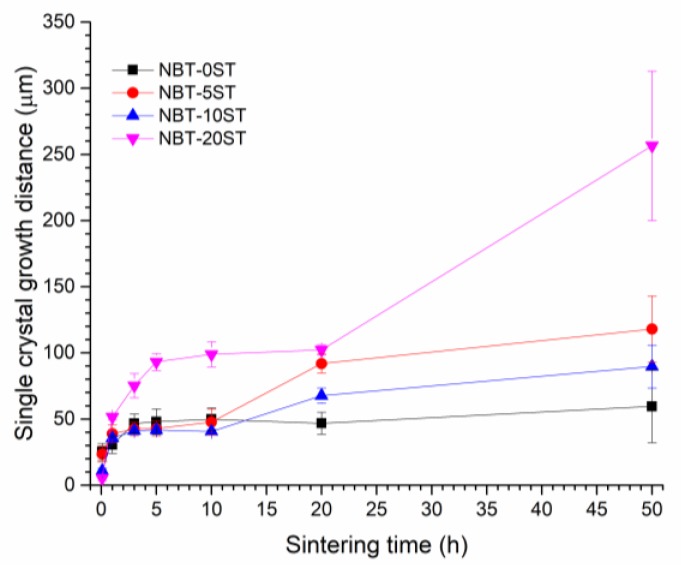
Mean single crystal growth distance of (1−x)(Na_1/2_Bi_1/2_)TiO_3_-xSrTiO_3_ samples as a function of sintering time.

Figure 6 shows single crystal growth distance vs. sintering time. Each data point is the mean value of 50 measurements of growth distance, with error bars representing standard deviation. For the NBT-0ST samples, the single crystal growth distance increases for sintering times up to 3 h, but after that growth levels off. For the NBT-5ST, NBT-10ST and NBT-20ST compositions, the behaviour is different. For the NBT-5ST and NBT-10ST compositions, single crystal growth distance increases up to 3 h, levels off between 3 and 10 h, increases again between 10 and 20 h, then starts to level off again between 20 and 50 h. For the NBT-20ST composition, growth rate increases up to 5 h, levels off between 5 and 20 h, then increases again between 20 and 50 h. The NBT-20ST composition shows the largest growth distance of all the compositions at all sintering times.

**Figure 7:**
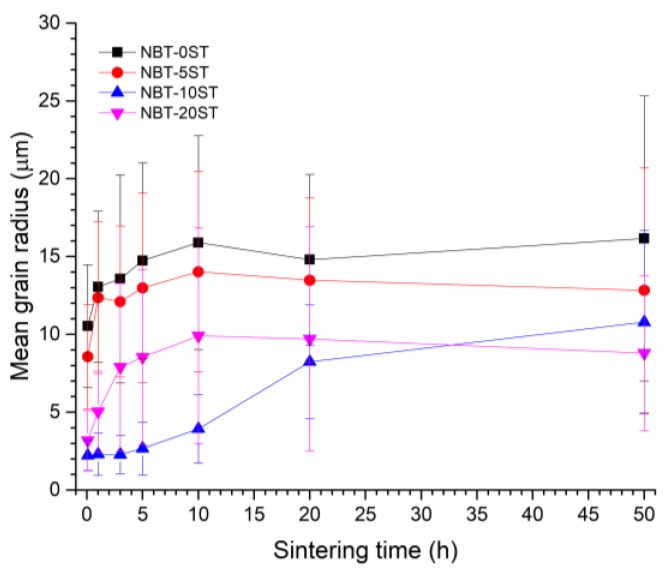
Mean grain size of (1−x)(Na_1/2_Bi_1/2_)TiO_3_-xSrTiO_3_ samples as a function of sintering time.

Mean matrix grain size increases with sintering time and changes with differing amount of SrTiO_3_ content as shown in Figure 7. To measure the size of a grain, the area of the grain is measured using the ParticleAnalyzer add-on for ImageJ and converted to an equivalent 2D spherical radius. Each data point is the mean value of at least 130 grains, with error bars representing standard deviation. For all compositions except NBT-10ST, matrix grain size increases with sintering time up to 10 h, after which grain growth levels off. For the NBT-10ST samples, grain growth is initially very slow, but increases after 5 h and continues up to 50 h. For the NBT-0ST, NBT-5ST and NBT-10ST samples, the mean grain size continuously decreases as the SrTiO_3_ content increases. Grain size increases again for the NBT-20ST samples.

**Figure 8:**
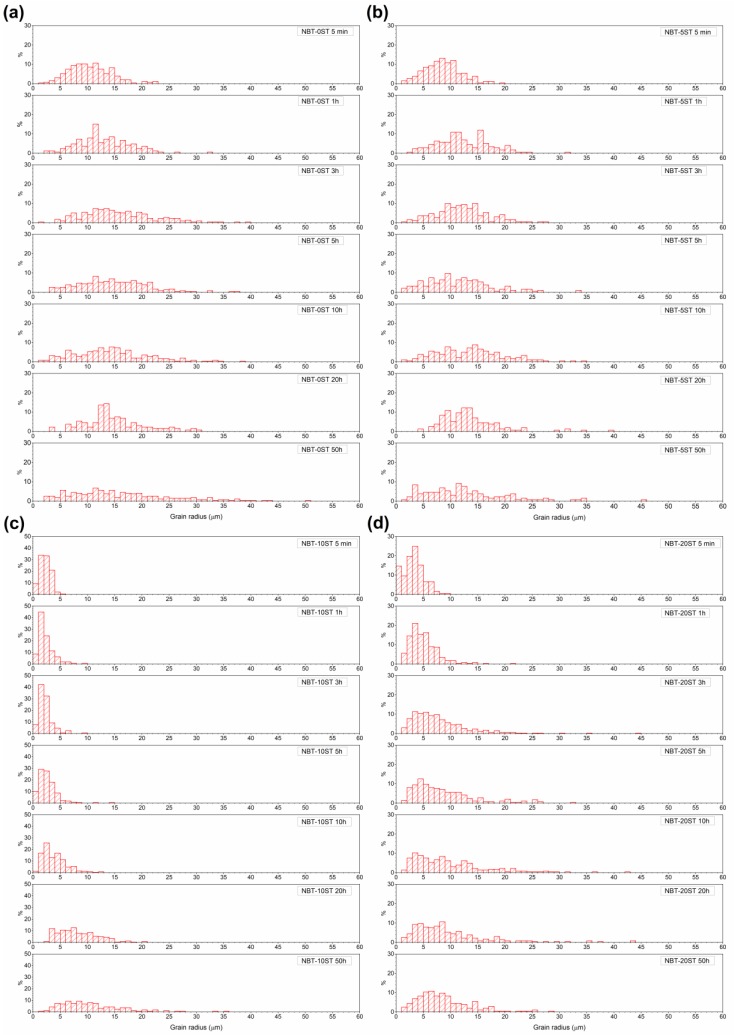
(**a**) NBT-0ST; (**b**) NBT-5ST; (**c**) NBT-10ST; (**d**) NBT-20ST grain size distributions as a function of sintering time.

Figure 8 shows matrix grain size distributions in the samples. The grain size distribution of the NBT-0ST sample is already quite broad after 5 min of sintering, with some abnormal grains present (defined as grains with a size ≥ twice the mean grain size). As sintering time increases, the grain size distribution becomes broader and more abnormal grains appear. For the NBT-5ST samples, the grain size distributions narrow slightly and the number of abnormal grains decreases. For the NBT-10ST samples the grain size distributions narrow dramatically, although abnormal grains are still present (note the different vertical scale). The distributions broaden slowly with sintering time and after 50 h the grain size distribution is similar to that of the other samples after 50 h, although not as broad. For the NBT-20ST samples, the behaviour reverses. The grain size distribution broadens after only 5 min of sintering. Further sintering causes the grain size distribution to broaden further and more abnormal grains to appear. However, the grain size distributions of the NBT-20ST samples remain narrower that the distributions of the NBT-0ST and NBT-5ST samples.

**Figure 9:**
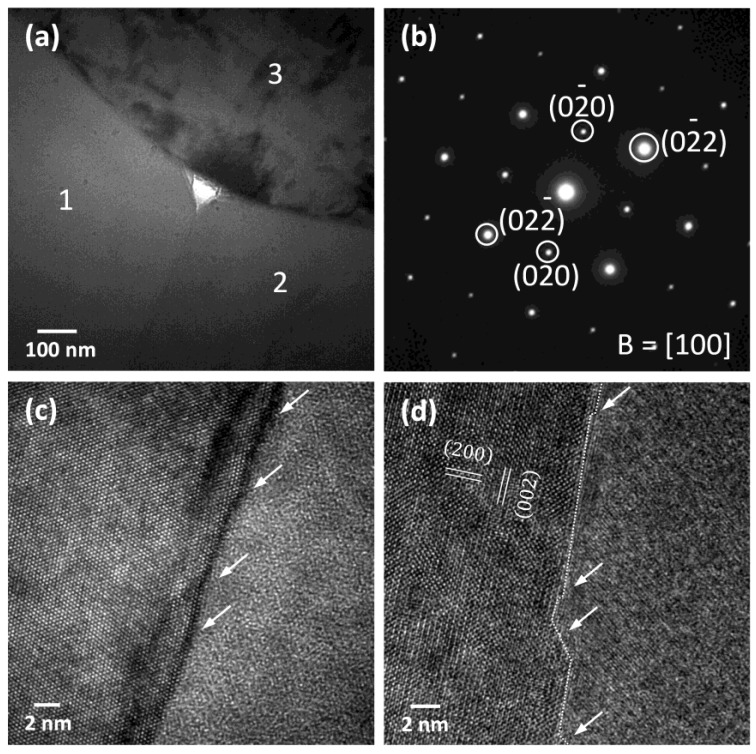
(**a**) TEM micrograph of NBT-0ST ceramic sample sintered at 1200 °C for 10 h; (**b**) diffraction pattern of the left hand matrix grain in Figure 9d; (**c**) HRTEM micrograph of the grain boundary between grains 1 and 2; (**d**) HRTEM micrograph of a grain boundary between two other grains not shown in Figure 9a.

Figure 9 shows TEM micrographs of an NBT-0ST ceramic sample sintered at 1200 °C for 10 h. Figure 9a is a micrograph of three matrix grains and a triple junction. The grains are labelled 1, 2 and 3. The grain boundary between grains 1 and 2 appears to be faceted, while the grain boundaries between grains 1 and 3 and grains 2 and 3 appear curved. The triple junction contains a secondary phase around a void caused by ion milling. Some of the interfaces between the matrix grains and the triple junction phase appear to be faceted and some appear to be curved. Figure 9b is a diffraction pattern of the left hand matrix grain in Figure 9d. Figure 9c is a high-resolution TEM (HRTEM) image of the grain boundary between grains 1 and 2. Microfacets are visible, marked with arrows. The HRTEM image of the grain boundary between grains 1 and 3 was not clear enough to determine if the boundary was faceted. Figure 9d is a grain boundary between two other grains not shown in Figure 9a. This grain boundary (marked with a dashed white line) is also faceted on an atomic scale.

**Figure 10:**
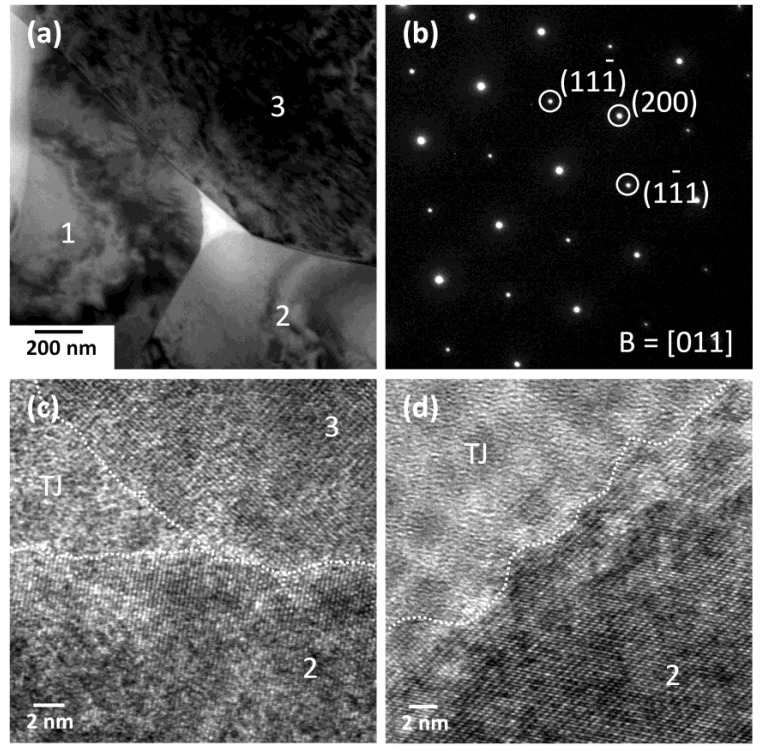
(**a**) TEM micrograph of NBT-10ST ceramic sample sintered at 1200 °C for 10 h; (**b**) diffraction pattern of grain 3 in Figure 10a; (**c**) HRTEM micrograph of grains 2 and 3 and the triple junction phase in Figure 10a; (**d**) HRTEM micrograph of grain 2 and the triple junction phase in Figure 10a.

Figure 10 shows TEM micrographs of an NBT-10ST ceramic sample sintered at 1200 °C for 10 h. Figure 10a shows a micrograph of three matrix grains and a triple junction (again the void in the triple junction phase is caused by ion milling). Microfaceting is visible at the interface between grain 1 and the triple junction phase. The interface between grain 2 and the triple junction phase is curved, whereas the interface between grain 3 and the triple junction phase has both faceted and curved regions. Figure 10b shows a diffraction pattern of grain 3. Figure 10c shows a HRTEM image of grains 2 and 3 and the triple junction phase, marked TJ. The interfaces between the grains and the triple junction phase appear rough, as does the grain boundary between the grains. Figure 10d shows a HRTEM image of the interface between grain 2 and the triple junction phase. The interface between the grain and the triple junction phase appears rough.

## 4. Discussion

Both single crystal and matrix grain growth in the (1−x) (Na_1/2_Bi_1/2_)TiO_3_-xSrTiO_3_ system depend on the structure of the solid/liquid interfaces or grain boundaries, which can be disordered (rough) or ordered (smooth/faceted) on the atomic level [24,32,33,34,35,36]. Interface structure in turn determines whether grain growth is diffusion-controlled or interface reaction-controlled. For a system with solid/liquid interfaces, the equation for the driving force Δ*G* for growth of a particular grain is [36,37,38]:

(1) $$\Delta G=2\gamma V_{m}\left(\frac{1}{\bar{r}}-\frac{1}{r}\right)$$

where *γ* is the solid/liquid interfacial energy, *V_m_* the molar volume, *r* the radius of the grain of interest and $\bar{r}$ the radius of a critical grain that is neither growing nor shrinking i.e., Δ*G* = 0. For a system with diffusion-controlled growth, $\bar{r}$ is the mean grain radius *r_mean_*, but for a system with interface-reaction controlled growth, $\bar{r}$ is smaller than *r_mean_* [38]. If Δ*G* is positive, then the grain can potentially grow. If Δ*G* is negative, then the grain can shrink. Because the seed crystal has *r* >>$\bar{r}$, the single crystal’s driving force for growth can be approximated by:

(2) $$\Delta G=\frac{2\gamma V_{m}}{\bar{r}}$$

**Figure 11:**
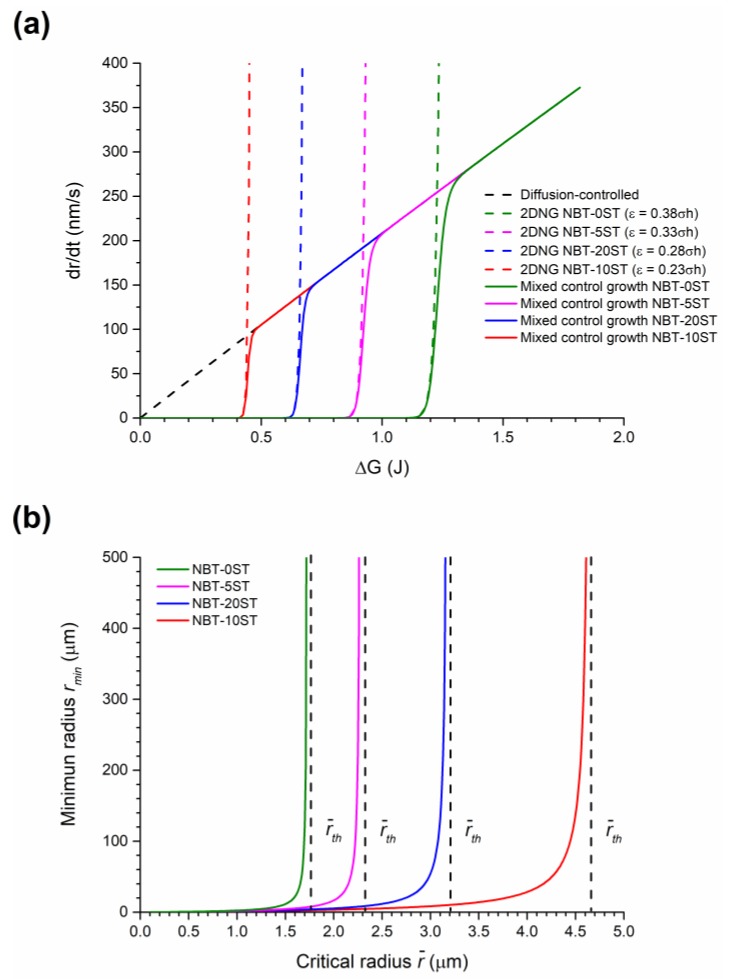
(**a**) Schematic relationship between grain growth rate (*dr/dt*) and driving force (Δ*G*) for different compositions in the NBT-100xST system; (**b**) schematic diagram of *r_min_* vs. $\bar{r}$ for different compositions in the NBT-100xST system. Note that this figure is only intended to describe the general changes in single crystal and grain growth behaviour as *ε* and Δ*G_c_* change. The values of *ε* for the different compositions in the NBT-100xST system are not known.

A grain in a system with disordered interfaces has an unlimited number of sites on its surface for atom attachment or detachment. The rate of atomic diffusion across the solid/liquid interface between the shrinking and the growing grain limits grain growth [36,37,39]. For a grain of radius *r*, growth rate is given by [40]:

(3) $$\frac{dr}{dt}\approx \nu_{D}=\frac{A}{r}\left(\frac{1}{\bar{r}}-\frac{1}{r}\right)\cdot \left(1+\beta \frac{r}{\bar{r}}\right)$$

where *ν_D_* is the diffusion-controlled growth rate, *A* = (*2γV_m_D_f_C*_∞_*/RT*) where *D_f_* = diffusion coefficient of solute atoms in the liquid, *C*_∞_ = solute concentration in the liquid for an infinitely large grain, *R* = the gas constant, *T* = absolute temperature and *β* is a function of solid volume fraction. Growth rate shows a linear increase with Δ*G* (the dashed black line in Figure 11a) and any grain with Δ*G* > 0 can grow. The relative size distribution of the grains ($r/r_{mean}$) does not change with sintering time and neither single crystal nor abnormal grain growth occur [34,35].

On the other hand, it is difficult for atoms to attach to the grain surface in a system with ordered interfaces. Because of a high number of broken bonds, such atoms are unstable and will detach from the grain unless they can attach to a low energy kink site such as a step formed by a two-dimensional nucleus or screw dislocation. Such sites are necessary for the grains to grow, which causes a nonlinear relationship between grain growth rate and Δ*G* [38,41,42]. For a system in which grain growth is controlled by nucleation and growth of 2D nuclei, the rate at which stable 2D nuclei form varies exponentially with Δ*G* [43,44,45]. Below a critical driving force Δ*G_c_*, the rate at which stable nuclei form is very low and hence grain growth is very slow. The rate at which stable nuclei form increases exponentially at Δ*G_c_*. Atoms reaching the grain can now easily find kink sites to attach to and the grain can grow. For 2D nucleation-controlled growth the grain growth rate is given by [37,41]:

(4) $$\frac{dr}{dt}\approx \nu_{R}=Bexp\;\left(-\frac{C}{\frac{1}{\bar{r}}-\frac{1}{r}}\right)$$

where *ν_R_* is the interface reaction-controlled growth rate. *B* = (*hFψn*_0_) where *h* = step height of the 2D nucleus, *F* = grain facet size and *n*_0_ = number density of atoms in the liquid. *ψ* = n*νexp(Δ*g_m_*/*kT*), where n* = the number of atoms in a position near to a critical 2D nucleus, *ν* = vibration frequency of atoms in the liquid, *k* = Boltzmann’s constant and Δ*g_m_* = activation energy for crossing the liquid/solid interface. *C* = (*πε*^2^/6*kTγ*), where *ε* = step free energy (the excess energy due to the presence of the step). The grain growth rate is almost zero below Δ*G_c_* and then increases rapidly at Δ*G_c_* (the coloured dashed lines in Figure 11a).

For Δ*G* > Δ*G_c_*, the grain surfaces undergo kinetic roughening [44,46,47]. Atoms can readily attach to the grain surface due to the high number of stable 2D nuclei, causing grain growth to become diffusion-limited. The grain growth behaviour can then be described by Equation (3). Therefore for a system with ordered interfaces, grain growth behaviour is controlled by interface reaction for Δ*G ≤ ΔG_c_* and by diffusion for Δ*G > ΔG_c_*, with the slower process governing the overall kinetics. Similar to the work of Wynblatt and Gjostein for the coarsening of metal particles in a vapour phase [48], the overall grain growth rate can then be expressed as [36,37,49,50]:

(5) $$\nu =\frac{\nu_{D}\nu_{R}}{\nu_{D}+\nu_{R}}$$

The grain growth rate increases exponentially at Δ*G* ≈ Δ*G_c_*, after which it increases linearly as Δ*G* increases further (the solid lines in Figure 11a). Grains with Δ*G* < 0 will shrink with a linear dependence to Δ*G*, as atoms have no energy barrier to detachment from each corner of the grain and multilayer dissolution can occur [36,37,41,43,48].

The value of Δ*G_c_* is given by [32,36,41]:

(6) $$\Delta G_{c}=\frac{\pi \varepsilon^{2}}{kTh}{\left(ln\psi n_{0}\right)}^{-1}$$

Δ*G_c_* varies with step free energy *ε*, which in turn varies with composition, sintering atmosphere, addition of dopants and sintering temperature [51,52,53].

This combination of interface-reaction controlled growth and diffusion-controlled growth has been called the mixed control theory of growth [36,37,50]. Figure 11a shows a diagram of the calculated growth rate vs. driving force for systems controlled by diffusion-controlled growth alone (Equation (3)), 2D nucleation-controlled growth alone (Equation (4)) and mixed control growth (Equation (5)). The calculations were made using the following values for physical constants [37]: *h* = 1.2 × 10^−10^ m, *γ* = 0.1 J/m^2^, *B* = 1.0 × 10^−25^, *D_f_* = 10^−9^ m^2^/s, C_∞_ = 10 at.%, *V_m_* = 10^−5^ m^3^ and *ε* = *Dhγ* where *D* is a constant. *β* was set to 25 assuming a solid volume fraction close to one [40]. *T* was set as 1473K and $\bar{r}$ was set as 1.0 × 10^−6^ m. The constant *A* was calculated to be 1.63 × 10^−20^ m^3^/s. By changing the value of *D*, the effect of changes in *ε* on the grain growth rate can clearly be seen. As *ε* increases, the value of Δ*G_c_* also increases, as expected.

If Δ*G* is replaced with Δ*G_c_*, then Equation (1) can be written in the following form [38]:

(7) $$\Delta G_{C}=2\gamma V_{m}\left(\frac{1}{\bar{r}}-\frac{1}{r_{min}}\right)$$

where *r_min_* is the minimum value of radius needed for a grain to be able to grow rapidly by 2D nucleation-controlled growth. If the radius of a grain falls below *r_min_*, then the value of Δ*G* for that grain drops below Δ*G_c_* and its grow rate becomes very slow. A diagram of *r_min_* vs. $\bar{r}$ for different values of Δ*G_c_* is shown in Figure 11b. Values of *γ* and *V_m_* are the same as before. The values of Δ*G_c_* were estimated from Figure 11a. As $\bar{r}$ approaches a certain value, called the threshold value ${\bar{r}}_{th}$, the value of *r_min_* increases rapidly towards infinity. Once $\bar{r}$ = ${\bar{r}}_{th}$, growth by 2D nucleation-controlled growth becomes impossible for grains of any size.

For a system where mixed control growth controls grain growth behaviour, several types of grain growth can occur, depending on the relative values of Δ*G_c_* and Δ*G_max_* (where Δ*G_max_* is the driving force of the largest grain in the system) [36,37,50]. If Δ*G_c_* << Δ*G_max_*, a large number of grains have Δ*G* > Δ*G_c_* and can grow. Such growth behaviour is called pseudo-normal, due to its similarity to normal grain growth. If Δ*G_c_* ≈ Δ*G_max_*, most of the matrix grains have Δ*G* < Δ*G_c_* and barely grow. A small number of grains, called abnormal grains, have Δ*G* ≥ Δ*G_c_* and can quickly grow, becoming much larger than their neighbouring matrix grains. Such growth behaviour is called abnormal grain growth. If Δ*G_c_* >> Δ*G_max_*, none of the grains have Δ*G* > Δ*G_c_* and grain growth barely takes place (stagnant grain growth). In SSCG, the seed crystal is large enough to have a driving force > Δ*G_c_* [27,54,55]. It acts like an abnormal grain, consuming the surrounding matrix grains to form a single crystal with the same chemical composition as the matrix. Abnormal grain growth can also take place in the matrix if grains large enough to have Δ*G* ≥ Δ*G_c_* exist.

The above mixed control grain growth model was developed for liquid phase sintered systems in which a liquid phase separates the solid grains. From the TEM micrographs it is seen that the grains in the present work have solid-solid grain boundaries and a liquid phase at the triple junctions and corner pockets between grains (Figure 9 and Figure 10). Similar grain growth behaviour to the above model has also been found to occur in systems with solid-solid grain boundaries [31,56,57,58,59,60,61,62,63,64,65]. It should be emphasized that Figure 11 is only intended to describe the general changes in single crystal and grain growth behaviour as *ε* and Δ*G_c_* change. The values of *ε* for the different compositions in the NBT-100xST system are not known.

The value of *ε* can be qualitatively determined from the shape of the grains [50,66,67]. The equilibrium crystal shape of the grains changes with changing *ε*, from spherical (*ε* = zero) to cubic with sharp corners and edges (high *ε*) [68,69,70]. Kang and Moon previously found that grains of composition 0.95(Na_1/2_Bi_1/2_)TiO_3_-0.05BaTiO_3_ have a cubic shape with faceted faces and rounded corners [71,72] indicating that the composition has a moderate value of *ε* [68,69]. Grain growth behaviour was initially pseudo-normal but changed to abnormal after extended sintering [71]. Our previous work on the NBT-25ST system showed that the grains have a cubic shape with flat faces and rounded edges and corners [23]. The microfaceting on the grain boundaries of the matrix grains in the NBT-100xST system shows that the boundaries are faceted (Figure 5). TEM observation also shows that the grains have faceted (atomically ordered) grain boundaries and both faceted and curved corners at the triple junctions, indicating a moderate value of *ε* (Figure 9 and Figure 10).

In the NBT-0ST samples, the moderate value of *ε* means a moderate values of Δ*G_c_* and low value of *r_min_* (Equation (6), Equation (7) and Figure 11). At the beginning of sintering, due to the fine powder size, Δ*G_c_* << Δ*G_max_*, many grains have Δ*G* > Δ*G_c_* and *r* > *r_min_* and so the samples initially show pseudo-normal growth behaviour. Matrix grain growth proceeds slowly and single crystal growth barely takes place. As grain growth proceeds, *r_mean_* and $\bar{r}$ increase, causing a reduction in Δ*G* for all matrix grains (Equation (1)), a reduction in Δ*G_max_* and an increase in *r_min_* (Equation (7) and Figure 11b). The value of Δ*G_c_* is not affected as it does not depend upon grain size (Equation (6)). Hence the number of grains with Δ*G* > Δ*G_c_* and *r* > *r_min_* decreases. As the values of Δ*G* and *r* for a particular grain drop below Δ*G_c_* and *r_min_*, its growth rate decreases rapidly (Figure 11). Eventually, Δ*G_c_* ≈ Δ*G_max_* and only a few grains are large enough to have Δ*G* ≥ Δ*G_c_* and *r* ≥ *r_min_*. These grains grow more rapidly than their neighbors and become abnormal grains, causing broadening of the grain size distribution (Figure 8). This has already happened by 5 min for the NBT-0ST samples, as abnormal grain growth has taken place and the grain size distribution is already broad. At the same time, the single crystal will also start to grow more rapidly than the surrounding matrix grains, as it is larger than even the largest matrix grain in the system. As abnormal grain growth finishes, there will be a sudden increase in *r_mean_* and $\bar{r}$ as most of the matrix grains have been replaced by the abnormal grains [37]. The increase in *r_mean_* reduces Δ*G* for the abnormally growing grains and their growth rate slows with further sintering. This has already happened by 3 h of sintering. As the grain size distribution broadens further the values of Δ*G* and *r* for the abnormally growing grains drop below Δ*G_c_* and *r_min_* and the abnormally growing grains can barely grow, causing *r_mean_* and the grain size distribution to stagnate (Figure 7 and Figure 8). The single crystal growth rate also decreases due to the increase in *r_mean_* and $\bar{r}$ (Equation (2)). Eventually, $\bar{r}$=${\bar{r}}_{th}$ and even the single crystal almost stops growing as its value of Δ*G* drops below Δ*G_c_* (Figure 6).

The change in single crystal and matrix grain growth behaviour with formation of a solid solution of (Na_1/2_Bi_1/2_)TiO_3_ and SrTiO_3_ can be explained by a decrease in *ε*. For the NBT-5ST samples *ε* decreases, causing both Δ*G_c_* and *r_min_* to decrease (Equation (5), Equation (7) and Figure 11). Figure 11a shifts to lower values of Δ*G* and Figure 11b shifts to higher values of $\bar{r}$. Δ*G_max_* is not affected by the change in *ε* (Equation (1)) although it may be affected by changes in the mean particle size and size distribution of the starting powder. The decrease in mean particle size for the NBT-5ST powder will cause an increase in the initial values of Δ*G* for all the grains (Figure 2). Again, Δ*G_c_* << Δ*G_max_* and initial grain growth is pseudo-normal. The lower values of Δ*G_c_* and *r_min_* means that there are more grains with Δ*G* > Δ*G_c_* and *r* > *r_min_* i.e., more grains which can grow. This limits the growth rate of both the matrix grains and the single crystal, as there are now more grains competing for the material made available by the shrinking grains. The time at which Δ*G_c_* ≈ Δ*G_max_* and the grain growth behaviour shifts from pseudo-normal to abnormal will also be delayed, due to the decrease in Δ*G_c_* and *r_min_*. Although abnormal grain growth has still started within 5 min of sintering, the grain size distribution is narrower than that of the NBT-0ST sample (Figure 8). The onset of rapid single crystal growth is likewise delayed. Finally, when abnormal grain growth does start, the range of values of Δ*G* at which Δ*G_c_* ≈ Δ*G_max_* will be lower than in the case of the NBT-0ST samples and so the abnormally growing grains and the single crystal will have lower growth rates than those of the NBT-0ST samples (Figure 11a). Hence both *r_mean_* and the single crystal growth distance for the NBT-5ST samples are lower (Figure 6 and Figure 7). The grain size distributions are also narrower (Figure 8). Again, as *r_mean_* and $\bar{r}$ increases the abnormal grains will stop growing and grain growth stagnates. The single crystal growth rate also decreases as $\bar{r}$ approaches ${\bar{r}}_{th}$ and the value of Δ*G* for the single crystal becomes ≈ Δ*G_c_*. However, after 10 h the single crystal growth rate increases again. After a period of slow growth, the single crystal may have grown large enough for its value of Δ*G* to be greater than Δ*G_c_* and its growth rate may increase again. This may be similar to the phenomenon of secondary abnormal grain growth [37]. In the region of Δ*G* ≈ Δ*G_c_*, growth rate changes drastically with even small variations of Δ*G*.

For the NBT-10ST samples *ε*, Δ*G_c_* and *r_min_* decrease still further, and the *r_min_* vs $\bar{r}$ curve shifts to yet higher values of $\bar{r}$. The increase in mean particle size for the NBT-10ST powder will also cause the initial values of Δ*G* for all the grains to decrease (Figure 2). Psuedo-normal grain growth takes place as before and the start of abnormal grain growth is further delayed. From the grain size distributions, abnormal grain growth has only just started after 1 h. Once abnormal grain growth does start, the growth rate of the abnormal grains is lower than for the NBT-0ST and NBT-5ST samples because of the lower value of Δ*G_c_* at which abnormal grain growth and single crystal growth starts. The grain size distributions broaden very slowly, unlike the NBT-0ST and NBT-5ST samples. As a result, *r_mean_* only increases gradually with sintering time (Figure 7). Because of the slow growth rate of the abnormal grains and the slow increase in *r_mean_*, grain growth continues in the NBT-10ST samples even up to 50 h. The growth rate of the single crystal is also reduced compared to the NBT-0ST and NBT-5ST samples, because of the lower value of Δ*G_c_* at which abnormal grain growth and single crystal growth starts.

For the NBT-20ST samples, the behaviour changes. The increase in *r_mean_* (Figure 7) and the broader grain size distributions (Figure 8) compared to the NBT-10ST samples implies an increase in *ε* and Δ*G_c_*. This causes the *r_min_* vs. $\bar{r}$ curve to shift to lower values of $\bar{r}$. The decrease in mean particle size for the NBT-5ST powder will also cause an increase in the initial values of Δ*G* for all the grains (Figure 2). Despite this, the increase in *ε* and Δ*G_c_* means that pseudo-normal grain growth can only take place for a shorter time before the driving force and radii of most of the grains drop below Δ*G_c_* and *r_min_*. Abnormal grain growth then takes place as before and the growth rate of the abnormal grains is also increased compared to the NBT-10ST samples due to the higher value of Δ*G_c_*. The mean grain size increases compared to the NBT-10ST samples and the grain size distributions are broader. The rapid completion of abnormal grain growth and increase in *r_mean_* causes the grain growth to stagnate after 10 h (Figure 7). From the matrix grain growth behaviour, the value of *ε* for the NBT-20ST sample is expected to lie between those of the NBT-5ST and NBT-10ST samples, but the single crystal growth distance of the NBT-20ST samples is much higher than that of the other samples. The reason for this behaviour is not yet understood.

The change in *ε* with composition is difficult to predict. *ε* has both enthalpy *h_s_* and entropy *s_s_* terms and may be written as *ε* = *h_s_* − T*s_s_* [70]. Formation of a solid solution would increase configurational entropy which would in turn increase *s_s_* and decrease *ε* [73,74]. Changes in composition will also affect interatomic bond strengths and specific surface energies, which would cause a change in *ε*. The increased Sr-O bond strength ($D_{298}^{0}$ = 426 kJ/mol) compared to Na-O ($D_{298}^{0}$ = 270 kJ/mol) and Bi-O ($D_{298}^{0}$ = 337 kJ/mol) bond strengths [75] will increase *h_s_*. Initially, formation of a solid solution is expected to cause *ε* to decrease, as the increase in the configurational entropy and the entropy term T*s_s_* is larger than the increase in *h_s_*. With further addition of SrTiO_3_, the increase in configurational entropy levels off [74]. The increase in *h_s_* outweighs the entropy term and *ε* increases. Changes in *ε* with formation of a solid solution have been noted in several systems [34,35,51,76,77,78].

The results show that the 0.8(Na_1/2_Bi_1/2_)TiO_3_-0.2SrTiO_3_ (NBT-20ST) composition shows the largest single crystal growth distance. This is beneficial, as this composition is the closest to the MPB in the (Na_1/2_Bi_1/2_)TiO_3_-SrTiO_3_ system of the compositions studied and may be expected to have the best piezoelectric properties. Comparing the present results with earlier work on the 0.75(Na_1/2_Bi_1/2_)TiO_3_-0.25SrTiO_3_ composition, it appears that a further increase in SrTiO_3_ content will cause single crystal growth to slow down again [23]. So the NBT-20ST composition may be the most suitable composition in terms of crystal growth ability.

## 5. Conclusions

Single crystals of composition (1−x)(Na_1/2_Bi_1/2_)TiO_3_-xSrTiO_3_ (NBT-100xST) with x = 0.00, 0.05, 0.10 and 0.20 were grown by the solid state crystal growth method and the effect of increasing SrTiO_3_ concentration on single crystal and matrix grain growth studied. Single crystal growth is initially rapid, then levels off with sintering time. For sintering times up to 10 h, single crystal growth rate decreases with SrTiO_3_ content up to x = 0.10, then increases again for x = 0.20. For longer sintering times, single crystal growth rate can increase again in the compositions with SrTiO_3_ addition, particularly for x = 0.20. SrTiO_3_ addition up to x = 0.10 causes matrix grain growth to decrease, with further addition of SrTiO_3_ causing matrix grain growth to increase again. The single crystal and grain growth behaviour has been explained using the mixed control grain growth model. The results show that the 0.8(Na_1/2_Bi_1/2_)TiO_3_-0.2SrTiO_3_ (NBT-20ST) composition shows the largest single crystal growth distance. 

## Figures and Tables

**Table 1:** Energy dispersive X-ray spectrometer (EDS) analysis of a 0.8(Na_1/2_Bi_1/2_)TiO_3_-0.2SrTiO_3_ sample sintered at 1200 °C for 10 h.

Element	Single Crystal (at. %)	Matrix (at. %)	Nominal (at. %)
Na	5.3 ± 1.7	5.6 ± 1.5	8.0
Bi	7.9 ± 0.7	8.5 ± 1.5	8.0
Ti	20 ± 0.0	20 ± 0.0	20.0
Sr	3.7 ± 0.7	3.7 ± 0.4	4.0
O	46.3 ± 5.8	43.3 ± 5.0	60.0
